# Retraction statement: Knockdown of lncRNA MNX1‐AS1 suppresses cell proliferation, migration, and invasion in prostate cancer

**DOI:** 10.1002/2211-5463.13508

**Published:** 2022-11-15

https://doi.org/10.1002/2211-5463.12611

The above article published online on 22 February 2019 in Wiley Online Library (wileyonlinelibrary.com), has been retracted by agreement between the Editor‐in‐Chief and John Wiley and Sons Ltd. The retraction has been agreed following an investigation based on allegations raised by a third party, which revealed that some images in this article are inappropriate duplications of images from previously published articles [1, 2]. Thus, the editors consider the conclusions of this manuscript substantially compromised.
